# Breast cancer risk level and prediction of tumor aggressiveness in the Athena Breast Health Network

**DOI:** 10.1007/s10549-025-07894-1

**Published:** 2026-01-28

**Authors:** Katherine Leggat-Barr, Tomiyuri Lewis, Jeffrey A. Tice, Elene Tsopurashvili, Rosalyn Sayaman, Paige Warner, Kathy Malvin, Leah Sabacan, Alexandra Perry-Solomon, Sarah Theiner, Irene Acerbi, Ann Griffin, Joseph McGuire, Vivian Lee, Alexander D. Borowsky, Wisdom Study and Athena Breast Health Network Investigators and Advocate Partners, Martin Eklund, Celia Kaplan, Robert A. Hiatt, Allison Stover Fiscalini, Karla Kerlikowske, Yiwey Shieh, Laura Esserman, Laura van’t Veer

**Affiliations:** 1 University of California, San Francisco, San Francisco, CA 94115 USA; 2 Stanford Medical Center, Stanford, CA 94305 USA; 3 University of California, Davis, Sacramento, CA 95817 USA; 4 Karolinska Institute, Stockholm, Sweden; 5 Weill Cornell Medicine, New York, NY 10065 USA; 6 Department of Laboratory Medicine, University California San Francisco, 2340 Sutter St.,, San Francisco, CA 94115 USA

**Keywords:** Risk asssessment, Breast screening, Prevention, Cohort study

## Abstract

**Purpose:**

Determine the association between the Breast Cancer Surveillance Consortium v2 model (BCSC) risk score and advanced and non-advanced invasive breast cancer (IBC).

**Methods:**

We estimated BCSC 5-year invasive breast cancer risk for 11,915 participants in a prospective screening cohort with median follow-up of 6.9 years prior to breast cancer diagnosis. Individuals in the top 25% by age of BCSC risk standard were considered high-risk, those in the bottom 75% low-risk.

We obtained cancer outcomes, including American Joint Committee on Cancer (AJCC) prognostic pathologic stage, from the San Francisco Mammography Registry and an institutional cancer registry. We examined the associations of BCSC risk scores with advanced (≥ AJCC prognostic stage II) and non-advanced (AJCC prognostic stage I) IBC using Fisher’s exact test and logistic regression.

**Results:**

Of 11,915 participants, 4,005 (34%) were high-risk. There were 254 incident IBC cases, of which 40 (16%) were advanced and 214 (84%) were non-advanced. The median 5-year BCSC risk score for women with and without IBC was 1.83% and 1.45%, respectively (*p* < 0.001). High BCSC risk among women diagnosed with breast cancer was associated with non-advanced cancer (OR = 2.25, 95% CI = 1.71–2.95, *p* < 0.0001), but not with advanced cancer (OR = 1.20, 95% CI = 0.63–2.29, *p* = 0.57) compared to women not diagnosed with breast cancer.

**Conclusion:**

High BCSC risk scores were associated with high rates of non-advanced IBC. As non-advanced cancers are more likely to be hormone receptor-positive, BCSC may optimally identify candidates for endocrine risk reduction.

**Supplementary Information:**

The online version contains supplementary material available at 10.1007/s10549-025-07894-1.

## Introduction

Approximately 240,000 women are diagnosed with breast cancer each year in the United States and 42,000 die of the disease [1]. Current guidelines, including the United States Preventive Services Task Force (USPSTF) [2], the American College of Radiology (ACR) [3], and the American Cancer Society (ACS) [4], offer conflicting screening recommendations, with differing definitions of high-risk and the corresponding screening strategies. Most women receive annual mammography recommendations [5–7], despite differences in level of risk, breast cancer biology, and prognosis.

Assessment of individual risk for breast cancer has advanced significantly with the development of breast cancer prediction risk models [8–10]. Each model evaluates a different combination of risk factors and is used in the clinic to help guide when to start screening, its frequency, the use of supplemental imaging like MRI, and recommendations for risk-reducing medications [11]. One well-validated model developed by the Breast Cancer Surveillance Consortium (BCSC) is commonly used in clinical practice [12]. It utilizes age, first-degree family history of breast cancer, BI-RADS breast density, prior biopsy history and benign breast disease result, and race and ethnicity to predict a woman’s 5- and 10-year risk for invasive breast cancer [8, 12]. It was developed in a cohort of over one million women undergoing mammography screening in the United States.

Although breast cancer risk models help inform breast screening guidelines and clinical interventions, most health care providers use them in the clinic today to predict the risk of developing breast cancer versus no cancer, rather than the subtype of breast cancer. This poses limitations as breast cancer is a heterogeneous disease, ranging from cancers with a very rapid growth to those that have an indolent trajectory, and likely presenting themselves either at advanced or non-advanced stage, respectively [13]. This difference in growth pattern and related stage at presentation thereby impacts the effectiveness of screening [14]. Differences in tumor biology also have important implications for the most appropriate risk-reducing and prevention strategies. For example, individuals at risk for slower-growing hormone receptor-positive cancers are more likely to benefit from endocrine risk-reducing medications [15], while those at risk for faster-growing cancers may benefit from more frequent screening and the use of supplemental imaging [16, 17]. An established way to identify the aggressiveness of breast cancer has been defined by the American Joint Committee on Cancer (AJCC) based on pathologic stage and tumor features, where prognostic pathologic stage II or higher is considered advanced [18, 19].

We analyzed the association between BCSC model version 2 risk and the occurrence of both advanced and non-advanced breast cancer in a cohort of women being regularly screened for breast cancer. Understanding whether a risk model predicts specific breast cancer types, such as advanced versus non-advanced disease, can better inform its use in risk assessment and prevention strategies.

## Methods

### Study cohort

Women enrolled between January 2012 and February 2022 in the Athena Breast Health Network (Athena) at the University of California, San Francisco (UCSF) who provided informed consent to use their health data for research purposes were included in this study (the Athena UCSF cohort). Athena unites the five University of California medical center breast clinics (UC San Diego, UC Davis, UC Los Angeles and UC Irvine) and the Sanford Health Network (serving North/South Dakota and Minnesota), with a goal of integrating research with clinical care to drive improvements in breast cancer screening and treatment [20]. Athena participants complete a health intake form at each mammogram appointment, which collects demographic information, personal health history, and family history. For this analysis, we used the UCSF data from each participant’s first completed mammography appointment intake form. The inputs used to calculate an individuals’ risk were collected prior to any breast cancer diagnoses.

We limited this analysis to those patients aged 35–74 years at study entry, with no previous diagnoses of DCIS and/or invasive breast cancer (IBC) or mastectomy, and who had available BI-RAD breast density information within 3 years of their intake form date. We used the information from the San Francisco Mammography Registry (SFMR), a breast imaging registry in the San Francisco Bay Area with 12 participating sites, aiming to better understand how breast imaging finds breast cancer in all women [21], to ascertain follow-up information for the Athena UCSF participants. Women missing one or more elements required for BCSC risk calculation or who were not included in the SFMR Registry were excluded from this analysis.

This Athena Breast Health Network Study was approved by the UCSF IRB.

### Risk calculation

Breast cancer risk was assessed using the BCSC-version 2 (BCSC) 5-year risk estimate for invasive breast cancer using the following inputs: age, race and ethnicity, first-degree family history of breast cancer, previous breast biopsies, biopsy result, and BI-RADS breast density [8]. Women were considered to have first-degree family history if their mother, sister, or daughter were diagnosed with DCIS or invasive breast cancer. Mammographic density was recorded using BI-RADS categories [22]. Breast biopsy categories include no prior biopsy, prior biopsy with unknown diagnosis, non-proliferative lesion, proliferative without atypia, proliferative with atypia, and LCIS. The Athena intake questionnaire permits patients to select from a subset of these responses – no prior biopsy, prior biopsy unknown, prior biopsy with unknown diagnosis, or prior biopsy proliferative with atypia.

### Defining high-risk

To account for the association between older age and on average an expected higher BCSC absolute 5-year risk score, we first stratified the Athena cohort for each year of age into percentiles with their respective absolute 5-year BCSC risk scores. Subsequently, the threshold to define high-risk of the Athena cohort was based on absolute risk by age for the top 25% as observed in the original BCSC model general population, which was considered the standard [12]. The top 25% of absolute risk by age represents a BCSC 5-year risk ranging from at least 0.44% for a 35-year-old to a 2.43% for a 74-year-old.

We also did a supplemental analysis classifying women in the top 2.5% by age as ‘high’ risk, which represents a BCSC 5-year risk ranging from at least 0.85% for a 35-year-old to a 3.93% for a 74-year-old.

### Breast cancer diagnoses

Cancer diagnoses were obtained through linkage with the SFMR Registry, which collects cancer outcome data from the California Cancer Registry (CCR) a component of the national SEER registry, and through linkage with the UCSF Cancer Registry, which includes breast cancer diagnoses for all UCSF patients. At the time of our linkage in 2022, SFMR/CCR cancer data were up to date through December 2018, and the UCSF Cancer Registry was up to date through January 2022.

### Definition of advanced stage cancer

Advanced stage cancer assessment followed the AJCC definition of prognostic pathologic stage II or higher. All other invasive cancers were considered non-advanced. This definition considers advanced breast cancers as a representation of aggressive tumors [23]. Pathologic features included tumor size, nodal status and metastases (TNM) and estrogen and progesterone hormone receptor (HR) status, human epidermal growth factor receptor 2 (HER2) status, and grade [23]. We chose to use pathologic prognostic staging, as it has been shown to have better prognostic value than anatomic staging [23].

### Statistical analysis

Person-years of follow-up for those who were not diagnosed with cancer were calculated using the date of the first Athena intake form completion through the most recent cancer registry information (January 2022). For those who were diagnosed with cancer, it was calculated from the date of first Athena intake form completion through their diagnosis date.

Mean 5-year BCSC v2 risk score was compared between those with invasive breast cancer and those without (reference population) by a two-sided *t*-test. In addition, we compared the mean 5-year BCSC scores among those who developed advanced cancer versus no cancer and those with non-advanced versus no cancer.

**Fig. 1:**
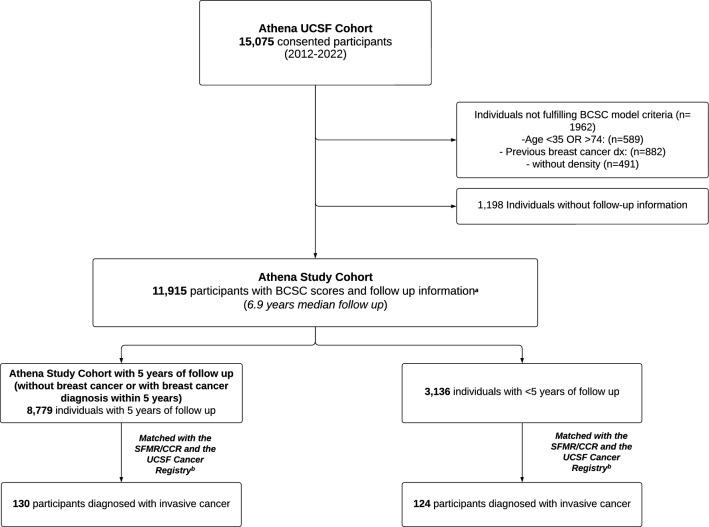
Athena UCSF Cohort Description with BCSC model risk scores and cancer diagnoses derived from the San Francisco Mammography Registry/California Cancer Registry (CCR) and UCSF Cancer Registry. ^a^ Complete BCSC inputs include all the following criteria: (1) 35–74 years old at the time of completing the first intake form; (2) not previously diagnosed with invasive breast cancer or DCIS; (3) available BI-RAD breast density information within 3 years of their first intake form. ^b^ Participants not found in SFMR/CCR either did not have breast cancer, were diagnosed outside of California, or were diagnosed between 2018 and 2022 when CCR data were incomplete. Participants not found in the UCSF Cancer Registry either did not have breast cancer or were not diagnosed/seen at UCSF between 2012 and 2022

To evaluate the 5-year BCSC risk model (expected) versus the occurrence of invasive breast cancer (observed), we included those women who had at least 5 years of follow-up since first completed Athena intake form and included only cancer diagnoses that occurred within 5 years (Fig. 1). The expected value was calculated as the 5-year cohort size times the mean value of all the individual risks as calculated for each subject by the BCSC model. Confidence intervals were calculated using Byar’s approximation as implemented in the `epi.smr` function in the `epiR` R package [24].

To estimate the odds of developing breast cancer versus no cancer, advanced IBC versus no cancer and non-advanced IBC versus no cancer, we performed several univariate logistic regressions. We estimated the odds ratio for developing those types of cancer versus no cancer in the full cohort within the top 25% versus the bottom 75% by age of BCSC risk with a significance level of 0.05. We performed the same analyses with the 2.5% by age threshold. We used the chi-squared and Fisher’s exact test (given small cell numbers) for proportions.

All analyses were performed using R Studio version 4.2.1 (R Foundation for Statistical Computing, Vienna Austria).

## Results

### Demographics

A total of 15,075 consented participants were identified as the Athena UCSF cohort (Fig. 1). 1962 individuals did not fulfill the BCSC model criteria: 589 were outside the age range (< 35 or > 74 years old), 882 had a prior breast cancer diagnosis, and 491 had missing BI-RAD breast density scores. 1198 were not found in the SFMR database and thus were without follow-up information. This resulted in the final Athena study cohort of 11,915 women (Fig. 1). The average age of the study cohort was 54.3 years, and the median follow-up was 6.9 years. Within this cohort, 254 (2%) developed invasive breast cancer (Table 1) over a period of 72,855 person-years.

**Table 1:** Characteristics of UCSF Athena cohort without or with invasive breast cancer

Total Athena Participants (N = 11,915)	No invasive breast cancer (N = 11,661)	With invasive breast cancer (N = 254)
Characteristics		
Average Age, years^†^	54.3	55.9
Race/ethnicity^†^		
American Indian, non-Hispanic	15 (0.1%)	0 (0%)
Asian, non-Hispanic	2,039 (17.5%)	42 (16.5%)
Black, non-Hispanic	494 (4.2%)	7 (2.8%)
Hispanic	1,053 (9%)	22 (8.7%)
Other/two or more races	759 (6.5%)	19 (7.5%)
White, non-Hispanic	7,301 (62.6%)	164 (64.6%)
First-degree family history of breast cancer^a†^	2,725 (23.4%)	93 (36.6%)
BI-RADS Breast density^b^		
Almost entirely fat	1,085 (9.3%)	20 (7.9%)
Scattered fibroglandular densities	4,228 (36.3%)	82 (32.3%)
Heterogeneously dense	5,059 (43.4%)	115 (45.3%)
Extremely dense	1,289 (11.1%)	37 (14.6%)
Biopsy results^†^		
Unknown prior biopsy	703 (6%)	9 (3.5%)
None (no prior biopsy)	8,096 (69.4%)	151 (59.4%)
Biopsy, non-specified benign	2,766 (23.7%)	88 (34.6%)
Atypical hyperplasia	96 (0.8%)	6 (2.4%)

^a^First-degree relatives defined as mother, sisters, and/or daughters

^b^BI-RADS = Breast Imaging Reporting and Data System

^†^Self-reported

Women who developed breast cancer were older and more likely to have first-degree family history; BI-RADs density category 4 (extremely dense) or a prior biopsy with an unknown diagnosis or atypical hyperplasia (Table 1), as compared to women with no cancer.

### BCSC predictions – observed versus expected

Among 8,779 women in the Athena study cohort with at least 5 years of follow-up and complete data, 130 were diagnosed with invasive breast cancer within 5 years (Fig. 1). With 129 cases expected, the observed/expected ratio was 130/129 (*p* = 0.99).

The mean 5-year BCSC score was significantly higher for women who developed breast cancer compared with those who did not (1.83% versus 1.45%, *p* < 0.001) (Supplemental Table 1A). Additionally, women stratified by increasing BCSC 5-year risk scores (< 1%, ≥ 1–3%, ≥ 3%) showed higher breast cancer incidence (Supplemental Table 1B). For example, in the category ≥ 3% BCSC 5-year risk, 14.1% of the women developed breast cancer in this category versus 6% of the women with no breast cancer.

### BCSC stratification by risk level and age

We dichotomized the 11,915 cohort into a high-risk group (participants in the top quartile of age-specific BCSC risk) (*n* = 4005) and a low-risk group (*n* = 7910) (Table 2). The proportion of the Athena cohort that fell into the top 25% of risk (*n* = 4005) is 34% of the total, indicating our population had higher average risk than the general population on which the BCSC model was validated [12]. In the Athena cohort, 254 participants developed invasive breast cancer. 51% participants who developed breast cancer (*n* = 128/254) were in the top 25% of age-adjusted risk compared to only 33% of the healthy controls (*n* = 3877/11,661), *p* < 0.001 (Table 2).

**Table 2:** The occurrence of breast cancer in the Athena cohort by BCSC risk stratum (top 25% vs bottom 75% by age)

	High Risk Top 25% BCSC score by age^a^ [*n* = 4005]	Low Risk Bottom 75% BCSC score) by age [*n* = 7910]	*p*-value^b^
No Cancer (control, *n* = 11,661)	3877 (33%)	7784 (67%)	reference
Invasive Breast Cancer (cases, *n* = 254)	128 (51%)	126 (49%)	*p* < 0.001

^a^The top 25% vs the bottom 75% BCSC risk by age threshold was based on absolute risk by age as observed in the original BCSC model population

^b^Fisher exact tests were run in comparison to women who did not develop breast cancer in the Athena cohort

Women in the top 25% of 5-year BCSC risk by age were significantly more likely to develop breast cancer than those in the bottom 75% of risk (OR = 2.04, 95% CI = 1.59–2.62, *p* < 0.0001) (Table 3; top row).

**Table 3:** The likelihood of developing breast cancer in the Athena cohort by BCSC risk stratum (top 25% vs bottom 75% by age)

Likelihood to develop breast cancer for the top 25% vs the bottom 75% of BCSC risk scores by age	Odds Ratio	95% Confidence Interval	*p*-value^a^
Invasive Breast Cancer vs no cancer	2.04	1.59–2.62	*p* < 0.0001
Non-advanced cancer vs no cancer (AJCC prognostic stage 1)	2.25	1.71–2.95	*p* < 0.0001
Advanced cancer vs no cancer (≥ AJCC prognostic stage 2)	1.20	0.63–2.29	*p* = 0.569

^a^Odds ratios were calculated in comparison to women who did not develop breast cancer in the Athena cohort by logistic regression

We observed similar results across high-risk cutoffs up to the top 2.5% of age-adjusted BCSC risk (OR = 2.53, 95% CI = 1.76–3.64, *p* < 0.0001) (Supplemental Table 2A and 2B).

### BCSC risk and cancer stage

Next, we evaluated if the BCSC risk was associated with predicting certain types of breast cancer. Among the 254 breast cancers, 214 (84%) were non-advanced stage and 40 (16%) were advanced stage (Table 4).

**Table 4:** The occurrence of non-advanced and advanced breast cancer by AJCC prognostic stage in the Athena cohort by BCSC risk stratum (top 25% vs bottom 75% by age)

	High Risk by Age Top 25% BCSC score [*n* = 4005]^a^	Low Risk by Age (Bottom 75% BCSC score) [*n* = 7910]	*p*-value^b^
No Cancer (control, n = 11,661)	3877 (33%)	7784 (67%)	
Non-advanced cancer (AJCC prognostic stage 1)	113 (53%)	101 (47%)	*p* < 0.001
Advanced cancer (≥ AJCC prognostic stage 2)	15 (37.5%)	25 (62.5%)	*p* = 0.6147​

^a^The top 25% vs the bottom 75% BCSC risk by age threshold was based on absolute risk by age as observed in the original BCSC model population

^b^Fisher exact tests were run in comparison to women who did not develop breast cancer in the Athena cohort

High risk women (top 25% risk by age) were more likely to develop non-advanced breast cancer than women in the bottom 75% of risk (OR = 2.25, 95% CI = 1.71–2.95, *p* < 0.0001) (Table 3; second row).

However, high-risk women (top 25% risk by age) were not more likely to develop advanced breast cancer than women in the bottom 75% of risk (OR = 1.20, 95% CI = 0.63–2.29, *p* = 0.569)., *p* < 0.001 (Table 3).

Specifically, 53% of the non-advanced cancers (*n* = 113/214) were in the top 25% of age-adjusted risk compared to 33% of healthy controls (3877/11,661) (*p* < 0.001) (Table 4). In comparison, 37.5% of the advanced cancers (*n* = 15/40) fell in the top 25% of BCSC age-adjusted risk compared to 33% of controls (*n* = 3877/11,661) (*p* = 0.5131) (Table 4).

Similar results were found using other risk thresholds up to the top 2.5% of age-adjusted BCSC risk for both non-advanced (OR = 2.78, 95% CI = 1.9–4.08, *p* < 0.0001) and advanced breast cancer types (OR = 1.28, 95% CI = 0.39–4.17, *p* = 0.678) (Supplemental Table 2B & 3).

When comparing the mean BCSC risk scores among women who developed advanced versus non-advanced types of breast cancer, we found similar results (Supplemental Table 1A). The mean 5-year BCSC scores were higher among those who developed non-advanced (1.84) compared to women without cancer (1.45) (*p* < 0.001). On the contrary, the mean BCSC scores between those who developed advanced (1.77) compared to women without cancer (1.45) were similar (*p* = 0.061).

## Discussion

The BCSC invasive cancer model was well-calibrated overall in the UCSF Athena Cohort. As expected, a higher 5-year BCSC risk was associated with higher rates of invasive breast cancer development. We found high-risk participants were not at significantly elevated risk for advanced cancers, despite the significant association with both invasive and non-advanced cancers. This suggests that the BCSC risk model preferentially predicts tumors with better prognosis, an important consideration that can inform decision-making around how this model is used to guide screening and prevention.

### Impact on clinical practice

Screening programs can utilize breast cancer prediction models to assess a woman’s level of risk for developing breast cancer to tailor screening recommendations and to identify women who may benefit from interventions to lower their risk for breast cancer. However, it is important to understand what type of breast cancer the model predicts to optimally guide clinical recommendations.

The BCSC model predicts non-advanced cancers, but not advanced cancers. This suggests that women with high 5-year BCSC risk would be more likely to benefit from endocrine risk-reducing medications such as tamoxifen, as non-advanced cancers are primarily hormone receptor-positive [25]. Over the past few decades after ASCO guidelines were released in 1999, prophylactic use of selective estrogen receptor modulators (SERMs) like tamoxifen have become more prevalent, especially for high-risk women [26]. SERMs act as “estrogen agonists in some tissues (bone, liver, and cardiovascular system) and antagonists in other tissues (breast and brain)” [27]. While the use of these medications prophylactically is shown to dramatically reduce the incidence of estrogen receptor (ER) positive cancers (sometimes by over 50%), they have been shown to have little effect on reducing the incidence of ER-negative cancers [15]. Our findings highlight an additional way to identify who is at risk for ER-positive breast cancer, which can facilitate and possibly increase the appropriate use of prophylactic SERMs.

Identifying women who are at high-risk for developing advanced breast cancer has only recently become a focus of risk prediction [17, 28]. It is women at risk for advanced breast cancer who would be more likely to benefit from increased screening frequency and consideration for supplemental imaging, since these cancers are faster-growing and often present themselves at later stage and/or as interval cancers [29, 30]. In addition, these tumors are more often HR negative and therefore endocrine-insensitive [25].

### Classification of ‘high risk’

In this paper, we examined two definitions of ‘high-risk’ women: those in the top 25% of risk by age and those in the top 2.5% by age. Defining the optimal high-risk is challenging because the threshold must be high enough to capture most cancer cases while preserving the model’s ability to distinguish between risk levels.

Currently, the Food and Drug Administration (FDA) uses a 5-year breast cancer risk of > 1.67% as the threshold at which high-risk individuals are approved for risk-reducing medications [31]. A 1.67% 5-year risk threshold captures 31% of our study population and 46% of cancers (data not presented in the manuscript). This makes our top 25% by age threshold comparable to the established FDA cutoff.

In contrast, USPSTF defines high-risk women as those with greater than 3% five-year risk of developing breast cancer, as these women are “likely to derive more benefit than harm from risk-reducing medications” [32]. A 3% 5-year risk captures 6% of our population, encompassing 14% of cancer diagnoses (data not presented in manuscript). This is comparable to our top 2.5% by age threshold, which was chosen as a high-risk threshold in the WISDOM trial [33]. This threshold consistently identifies women with a high lifetime risk of 23–28%, translating to a high enough risk where women would often consider using endocrine risk-reducing therapy [34]. While this high-risk threshold maintains strong discrimination with a high specificity, it misses most cancer cases due to the fact it only captures a very small proportion of women.

The ideal high-risk cutoff should balance maximizing benefits for high-risk women while minimizing harm to lower-risk women. Setting the threshold too low can increase screening program costs without providing additional benefit to women.

### Study limitations

Even though the BCSCv2 model was well-calibrated in our cohort with advanced and non-advanced cancer proportions of 214 non-advanced and 40 advanced stage cancer diagnoses, the results will be strengthened with longer follow-up and a higher number of breast cancers diagnosed. Our study was performed at a single center and has small numbers of American Indian, Black/African American, and Hispanic individuals which limits the generalizability of our results. Another limitation is that the UCSF Athena cohort seems to be higher risk than general population as 34% (rather than expected 25%) fall in the top 25% of risk by age. This may reflect a more screening-engaged population, introducing potential selection bias that is skewed toward higher baseline risk. However, the BCSC model’s close calibration in this dataset and the consistent stage-specific differences (non-advanced vs advanced) support the robustness of our findings, despite these cohort characteristics. Additionally, screening frequency was not incorporated into our analysis. Differential screening frequencies could impact our results, as women who undergo more frequent screening are more likely to have more indolent cancers diagnosed. However, all women in this study were likely being regularly screened for breast cancer as part of their participation in the Athena Breast Health Network. Death follow-up data were not collected, which also could impact the validity of our results. As follow-up time is extended more cancers will be diagnosed and our statistical power will increase to improve confidence in our findings.

### Subtype-specific risk models

Current work is being done to refine breast cancer risk models to better understand who is at risk for what type of cancer. It would be preferred if these risk models not only predicted breast cancer risk broadly but also considered the aggressiveness of specific types of breast cancer.

The BCSC-version 2 model accurately predicts who is at increased risk for developing breast cancer [8], and in this study, we show that it preferentially predicts those at risk for non-advanced, slower-growing cancers. The BCSC consortium recently developed a 6-year cumulative advanced breast cancer risk model that specifically identifies individuals who are at risk for AJCC pathologic prognostic stage II or higher cancer, which often present themselves as interval cancers with more aggressive features [17]. The BCSC 6-year advanced model incorporates body mass index (BMI) and menopausal status, and reports risk by screening frequency alongside the original five risk factors and can help inform clinical decisions around routine screening and supplemental imaging.

Another type of risk tool used are polygenic risk scores (PRS), based on single nucleotide polymorphisms (SNPs) associated with breast cancer risk, of which the PRS-313 is frequently used [35]. Recent evaluation has found that an increase in PRS-313 is associated with favorable tumor characteristics, such as lower grade, hormone receptor positive status [36], as observed in less-aggressive tumor types.

However, more recently, researchers have constructed a PRS for Risk of Recurrence weighted on Proliferation (ROR-P), using SNPs and tumor gene expression data. This score is associated with worse survival and better captures aggressive tumor types [37].

Differences between the BCSC and PRS risk models spotlight how each can help identify women at risk for different types of breast cancers. These can more accurately inform the type of risk-reducing strategies offered to patients at elevated risk of both advanced and non-advanced cancers.

Current work in the WISDOM Study is focused on developing and incorporating new models that stratify risk by breast cancer subtype and calibrate for race/ethnicity background to advance personalized screening programs. The second iteration of WISDOM (WISDOM 2.0) specifically aims to understand who is at risk for developing aggressive breast cancer (www.thewisdomstudy.org).

## Conclusion

Our study found that the BCSCv2 model strongly predicts for non-advanced breast cancers, which suggests it could be used to optimally guide the use of preventive interventions. However, it does not predict advanced breast cancer, which limits its utility for guiding screening frequency and supplemental imaging.

**Wisdom Study and Athena Breast Health Network Investigators and Advocate Partners**

Laura Esserman, MD, MBA, University of California, San Francisco

Laura van ‘t Veer, PhD, University of California, San Francisco

Robert Hiatt, PhD, University of California, San Francisco

Jeff Tice, MD, University of California, San Francisco

Elad Ziv, MD, University of California, San Francisco

Amie Blanco, CGC, University of California, San Francisco

Barry Tong, CGC, University of California, San Francisco

Katherine Ross, CGC, University of California, San Francisco

Allison Fiscalini, MPH, University of California, San Francisco

Maren Scheuner-Purcell, MD, MPH, University of California, San Francisco

Kimberly Badal, PhD, University of California, San Francisco

Kim Rhoads, MD, University of California, San Francisco

Celia Kaplan, PhD, University of California, San Francisco

Christina Yau, PhD, University of California, San Francisco

Rashna Soonavala, BS, University of California, San Francisco

Katherine Leggat-Barr, BS, University of California, San Francisco

Tomiyuri Lewis, BS, University of California, San Francisco

Patricia Choy, MPH, University of California, San Francisco

Steffanie Goodman, MPH, University of California, San Francisco

Leah Sabacan, MS, University of California, San Francisco

Kenneth Wimmer, MD, University of California, San Francisco

Kelly Adduci, MPH, University of California, San Francisco

Natalie Kim, BS, University of California, San Francisco

Taylor Glatt, BS, University of California, San Francisco

Tianyi Wang, University of California, San Francisco

Advika Verma, BS, University of California, San Francisco

Jennifer Atamer, BS, University of California, San Francisco

Alondra Torres, BS, University of California, San Francisco

Irene Acerbi Soto, PhD, University of California, San Francisco

Kelly Blum, MS, University of California, San Francisco

Stephanie Flores, BS, Kannact

Roxanna Firouzian, MPH, Wildflower Health

Arash Naeim, MD, University of California, Los Angeles

Neil Wenger, MD, University of California, Los Angeles

Carlie Thompson, MD, University of California, Los Angeles

Antonia Petruse, MS, University of California, Los Angeles

Annette Stanton, PhD, University of California, Los Angeles

Alyssa Rocha, BA, University of California, Los Angeles

Liliana Johansen, BA, University of California, Los Angeles

Xochil Calderon, MPH, University of California, San Francisco

Alexander Borowsky, MD, University of California, Davis

Skye Stewart, MPH, University of California, Davis

Samrrah Raouf, University of California, Davis

Lydia Howell, MD, University of California, Davis

Hoda Anton-Culver, PhD, University of California, Irvine

Hannah Lui Park, PhD, University of California, Irvine

Deborah Goodman, MD, PhD, University of California, Irvine

Lisa Madlensky, PhD, University of California, San Diego

Andrea LaCroix, PhD, University of California, San Diego

Barbara Parker, MD, University of California, San Diego

Tracy Layton, MS, University of California, San Diego

Michael Hogarth, MD, University of California, San Diego

Sheri Hartman, PhD, University of California, San Diego

Diana DeRosa, CGC, University of California, San Diego

John Pierce, PhD, University of California, San Diego

Paloma Sales, PhD, San Francisco VA Health Care System, San Francisco, CA

Andrea Kaster, MD, Sanford Health

Jan Wernisch, BSN, Sanford Health

Larissa Risty, LCGC, Sanford Health

Olufunmilayo Olopade, MD, University of Chicago

Dezheng Huo, PhD, University of Chicago

Brenda Gonzalez, University of Chicago

Rachael Lancaster, MD, University of Alabama Birmingham

Le’Andrea Anderson, University of Alabama at Birmingham

James Esserman, MD, Diagnostic Center of Miami

Isabella Cabaleiro, MS, Diagnostic Center of Miami

Vignesh Arasu, MD, PhD, Kaiser Permanente Division of Research

Martin Eklund, PhD, Karolinska Institutet

Yiwey Shieh, MD, Weill Cornell Medicine

Karen Sepucha, PhD, Mass General

Vivian Lee, MS, WISDOM Advocate

Diane Heditsian, BS, WISDOM Advocate

Susie Brain, BS, WISDOM Advocate

Dolores Morehead, MS, APCC, WISDOM Advocate

## Supplementary Information

Below is the link to the electronic supplementary material.

Supplementary file1 (DOCX 19 KB)

## Data Availability

Data were generated by the authors but are not publicly available, however summary statistics may be provided upon request to the study investigators, under terms set forth by the study’s institutional review board. We are committed to collaboration as a large multi-site program and welcome future collaborations. Please contact the corresponding authors for further information on collaborating with the Athena team.
