# Supplementary material for: Breast cancer risk level and prediction of tumor aggressiveness in the Athena Breast Health Network
Source: Breast Cancer Res Treat. 2026 Jan 28;215(3):65. doi: 10.1007/s10549-025-07894-1 (PMC12852213; doi:10.1007/s10549-025-07894-1)
Supplement: Supplementary file 1 — Supplementary file1 (DOCX 19 KB) [file 10549_2025_7894_MOESM1_ESM.docx]

**Supplemental Table 1A:** Mean 5-year BCSC risk scores for women in the Athena cohort (n=11,915) without cancer or, with non-advanced or advanced cancer by AJCC prognostic stage

|  | **Mean 5-year BCSC score** | **p-value** ^a^ |
| --- | --- | --- |
| **No cancer** | 1.45 | Reference |
| **Invasive Breast Cancer** | 1.83 | P<0.001 |
|  | | |
| **Non-advanced cancer**  (AJCC prognostic stage 1)  (n=214) | 1.84 | P<0.001 |
| **Advanced cancer**  (≥ AJCC prognostic stage 2)  (n=40) | 1.77 | P=0.061 |

^a^ p values calculated by t-test

**Supplemental Table 1B** Athena cohort BCSCv2 5-year risk distribution and breast cancer status

| ​ | BCSC 5-year risk distribution  [n=11,915] | | |
| --- | --- | --- | --- |
|  | <1% ​  No. (%) ​ | ≥1-3% ​  No. (%) ​ | ≥3% ​  No. (%) ​ |
| Breast Cancer​  (n=254) | 58  (22.8%) ​ | 160  (63%) ​ | 36  (14.1%) ​ |
| No Breast Cancer  (n=11,661)​ | 4002  (34.3%) ​ | 6965  (59.7%) ​ | 694  (6.0%) ​ |

**Supplemental table 2A** The occurrence of breast cancer in the Athena cohort by BCSC risk stratum (top 2.5% vs. bottom 97.5% by age)

| **Likelihood to develop breast cancer for the top 2.5% vs the bottom 97.5% of BCSC risk score by age** | **High Risk by Age**  **(Top 2.5% BCSC score)**  **​[n=728]​^a^** | **Low Risk by Age**  **(Bottom 97.5% BCSC score)**  **[n=11,187]​** | **p-value^b^** |
| --- | --- | --- | --- |
|  | | | |
| **No Cancer**​  (control, n=11,661) | 693 ​  (6%)​ | 10968 ​  (94%)​ | ​reference |
| **Invasive Breast Cancer**​  (cases, n=254)​ | 35​  (13%)​ | 219​  (86%)​ | ​ p<0.001 |

^a^ The top 2.5% vs the bottom 97.5% BCSC risk by age threshold was based on absolute risk by age as observed in the original BCSC model population

^b^ Fisher exact tests were run in comparison to women who did not develop breast cancer in the Athena cohort

**Supplemental Table 2B** The likelihood of developing invasive breast cancer in the Athena cohort by BCSC risk stratum (top 2.5% of BCSC risk by age vs bottom 97.5%)

|  | **Odds Ratio^a^** | **Confidence Interval** | **p-value** |
| --- | --- | --- | --- |
|  | | | |
| **Invasive Breast Cancer** | 2.53 | 1.76- 3.64 | p<0.0001 |
|  | | | |
| **Non-advanced cancer vs. no cancer**  (AJCC prognostic stage 1) | 2.78 | 1.90-4.08 | p<0.0001 |
| **Advanced cancer vs. no cancer** (≥ AJCC prognostic stage 2) | 1.28 | 0.39-4.17 | p=0.678 |

^a^ Odds ratios were calculated in comparison to women who did not develop breast cancer in the Athena cohort by logistic regression

**Supplemental Table 3** The occurrence of non-advanced and advanced breast cancer by AJCC prognostic stage in the Athena cohort by BCSC risk stratum (top 2.5% vs. bottom 97.5% by age)

| **​** | **High Risk by Age**  **(Top 2.5% BCSC score)**  **​[n=728]​ ^a^** | **Low Risk by Age**  **(Bottom 97.5% BCSC score)**  **[n=11,187]​** | **p-value ^b^** |
| --- | --- | --- | --- |
|  | | | |
| **No Cancer**​  (control, n=11,661) ​ | 693 ​  (6%)​ | 10968 ​  (94%)​ | ​reference |
|  | | | |
| **Non-advanced cancer**​  (AJCC prognostic stage 1)  (n=214) ​ | 32  (15%)​ | 182​  (85%)​ | p<0.001​ |
| **Advanced cancer**​  (≥ AJCC prognostic stage 2) ​  (n=40) ​ | 3 ​  (7.5%)​ | 37 ​  (92.5%)​ | 0.5131 ​  ​ |

^a^ The top 2.5% vs the bottom 97.5% BCSC risk by age threshold was based on absolute risk by age as observed in the original BCSC model population

^b^ Fisher exact tests were run in comparison to women who did not develop breast cancer in the Athena cohort
